# Metacognitive Awareness Scale, Domain Specific (MCAS-DS): Assessing Metacognitive Awareness During Raven’s Progressive Matrices

**DOI:** 10.3389/fpsyg.2020.607577

**Published:** 2021-01-06

**Authors:** John H. H. Song, Sasha Loyal, Benjamin Lond

**Affiliations:** Division of Psychology, School of Applied Social Sciences, De Montfort University, Leicester, United Kingdom

**Keywords:** intelligence, metacognition, Raven’s Progressive Matrices, scales, IQ

## Abstract

Metacognition, the cognition about cognition, is closely linked to intelligence and therefore understanding the metacognitive processes underlying intelligence test performance, specifically on Raven’s Progressive Matrices, could help advance the knowledge about intelligence. The measurement of metacognition, is often done using domain-general offline questionnaires or domain-specific online think-aloud protocols. This study aimed to investigate the relationship between metacognitive awareness and intelligence via the design and use of a novel Meta-Cognitive Awareness Scale – Domain Specific (MCAS-DS) that encourages reflection of task strategy processes. This domain-specific scale was first constructed to measure participants’ awareness of their own metacognition linked to Raven’s Progressive Matrices (SPM). Following discriminatory index and Exploratory Factor Analysis, a 15-item scale was derived. Exploratory Factor Analysis showed five factors: Awareness of Engagement in Self-Monitoring, Awareness of Own Ability, Awareness of Responding Speed/Time, Awareness of Alternative Solutions and Awareness of Requisite Problem-Solving Resources. The intelligence level of ninety-eight adults was then estimated using Raven’s Standard Progressive Matrices. Participants also completed the MCAS-DS, and further items that examined their test-taking behavior and Confidence level. Metacognitive awareness was positively correlated to standardized IQ scores derived from the SPM whilst Over-Confidence derived using the Confidence level measure was negatively correlated to SPM. Despite some limitations, this study shows promise for elucidating the relationship between metacognitive awareness and intelligence using the task-specific scale.

## Introduction

Intelligence is a higher order cognition associated with metacognition (Swanson, 1992; Veenman and Beishuizen, 2004). In Sternberg’s (1988) Triarchic Model of Intelligence, metacognitive processes form an integral part. Metacognition refers to the human ability to reflect upon our own perceptions, thoughts, and actions (Valk et al., 2016; Sternberg, 2018); and is therefore, broadly defined as cognitions about cognitions, or thinking about one’s own thinking (Roberts and Erdos, 1993; Georghiades, 2004). Flavell (1976) described metacognition as “the active monitoring and consequent regulation and orchestration” of cognitive processes (p.232) and proposed that the two components of metacognition, knowledge and metacognitive experience, interact in monitoring and regulating cognitive processes (Flavell, 1988). Metacognitive monitoring and metacognitive control are processes related to information flow between two levels, the object- and meta-hierarchical levels, as discussed within a Metacognitive Model proposed by Nelson (1996). The object-level is a lower-level cognition, such as deriving a solution to a problem, which can itself be the subject of a higher meta-level cognition such as thinking about whether all the information required to derive the solution is available (Nelson, 1996). This 2-level (object- and meta-hierarchical levels) system can be extended to a 3-level metacognitive system where the mid-level cognition can both receive monitored information from the lowest level and itself being subject to the highest third level cognition’s control (Narens et al., 1996). There are other models proposed, including single, dual or hierarchical models which described the different way information- processing channels can lead towards task performance and subjective, confidence ratings, for example (Maniscalco and Lau, 2016). Others have proposed first-order, post-decisional and second-order models in an attempt to account for the relationship between self-evaluations of one’s own performance and their actual performance (Fleming and Daw, 2017).

Research suggests that metacognition is strongly related to problem-solving (Lucangeli et al., 1997), comprehension (Veenman and Beishuizen, 2004), memory (Schwartz et al., 2004), and learning (Veenman and Spaans, 2005; Stankov and Kleitman, 2014); consequently, research addressing educational needs has sought to study learning and memory related metacognition for quite some time. Conversely, meta-reasoning, defined as the monitoring of processes relating to more complex cognitive tasks such as problem-solving, has received far less attention until recently (Ackerman and Thompson, 2017). This monitoring can entail the regulation of time and effort allotted to a task (Ackerman and Thompson, 2017). It has been argued that qualitative differences between individuals in the *way* they effectively problem-solve is more pertinent than how *much* they engage in problem-solving (Evans, 2007). It is therefore important to examine the way individuals engage in reasoning. A few key research questions in meta-reasoning have been raised, including one that pertains to the current investigation, that being: “How do individuals differ in their ability to assess their performance?” (Ackerman and Thompson, 2017). Inherent in this question is the assumption that this partly relies on the individuals’ awareness of their own meta-cognitive processes. Not only do individuals rely on cues such as perceived ease of responding in monitoring their own performance, there are differences in the efficacy of the cues. Therefore, some of this information can be misleading (Ackerman and Thompson, 2017).

Whilst the relationship between metacognition and intelligence is not entirely clear (Stankov and Kleitman, 2014), empirical studies have shown a positive relationship between the two (Swanson, 1992; Veenman and Beishuizen, 2004; Veenman et al., 2014), suggesting that metacognitive awareness processes may vary when performing cognitive tasks. Metacognitive awareness of cognitive processes are more apparent in individuals who excel in cognitive activities than those who perform less well (Livingston, 2003). This includes intellectually gifted children, who display higher metacognitive knowledge than children of high-average and low-average intelligence (Swanson, 1992). At the other end of the intellectual spectrum, individuals with an intellectual disability may struggle to generate cognitive strategies and generalize the use of learned strategies to solve reasoning tasks such as Raven’s Standard Progressive Matrices (SPM) items (Campione and Brown, 1978). In view of such research, it has been posited that highly intelligent individuals may have additional cognitive resources with which to engage the task at hand as well as managing metacognitive activity (Ohtani and Hisasaka, 2018). Although the suggestion that the availability and utilization of extra cognitive resources by intelligent individuals remains speculative, it is supported by brain-imaging studies that show differential patterns of neurological activity among individuals of different ability levels when undergoing Raven’s Progressive Matrices (Song, 2005). This conforms to the neural efficiency hypothesis (Dunst et al., 2014), and Parieto-Frontal Integration Theory (Jung and Haier, 2007).

The relationship between metacognition and task performance varies according to the type of cognitive task being performed (van der Stel and Veenman, 2008). Raven’s Progressive Matrices has often been used as a measure of problem-solving ability, or *g*, general intelligence (Penrose and Raven, 1936), having been designed as a way to assess and measure eduction processes that Spearman emphasized as the fundamental nature of intelligence (Raven and Raven, 2003). A version of the task, the Standard Progressive Matrices (SPM), has been described as “one of the purest and best measures of *g* or general intellectual functioning available” (Raven et al., 2000). Although this has been contested, it is still often thought to measure the fluid intelligence branch within the Cattell-Horn-Carroll intelligence model (Stankov, 2000; Gignac, 2015; Waschl et al., 2016). Some have called it a “hallmark fluid intelligence test (Chuderski et al., 2020).

Rule induction and goal management are required to solve Raven’s Progressive Matrices (Loesche et al., 2015), and its utility in easily assessing cognitive ability has seen this reasoning task being used within several neuroimaging studies (Song, 2005; Chen et al., 2017; Duncan et al., 2017). In order to study the processes underlying Raven’s Progressive Matrices, a recent study examined participants who were required to self-monitor, remember their cognitive operations and appraise their solution. Participants’ subjective experiences were also measured as part of metacognition measurement. The results showed that the metacognitive experiences may vary within the set of matrices. The study demonstrated and argued that Raven’s Progressive Matrices is worthy of greater scrutiny due to its popularity as an intelligence test used in basic, educational and professional settings as well as the role it can play to advance the knowledge about human cognitive ability (Chuderski et al., 2020).

Therefore, given the close relationship between SPM and fluid intelligence, and the opportunity afforded by Raven’s Progressive Matrices in elucidating higher level cognitive processes, it is chosen as the focal task upon which metacognition, specifically, metacognitive awareness, will be measured in this study.

Measures of metacognitive performance risk being affected by confounding variables such as the nature of task demands alongside broader measurement difficulties; indeed, a key challenge in this area of research continues to be the availability of reliable measures for metacognitive ability (Kelemen et al., 2000). There exists metacognitive measures such as the Metacognitive Awareness Inventory (Schraw and Dennison, 1994). However, for reasons to be explained later, these are not suitable for the current investigation. Furthermore, there is the assertion that the type of metacognitive measure employed may moderate the metacognition-intelligence relationship (Ohtani and Hisasaka, 2018). Even the elicitation of participants’ responses to metacognition measures will alter the processes for which the measures were constructed (Double and Birney, 2019). Together, these make the accurate and valid measure of metacognition, an important issue for this field.

Existing measures do not always reflect the aspects of cognition involved in specific tasks such as intelligence tests. In other words, existing measures are too generic, instead of being specific which will measure individuals when they engage in metacognitive processes specific to a focal task. Some of the existing measures were constructed in order to apply them to a general learning context, and to specific populations such as children. This lack of specificity issue was raised by Allon et al. (1994), who in view of their own research yielding a non-significant result concerning the relationship between intelligence and metacognition, argued for the need for metacognitive measures to reflect the task demand of any corresponding intelligence test. A recent meta-analytic review continued to highlight the importance of considering the type of metacognition measurement used (Ohtani and Hisasaka, 2018). There are two main types of metacognitive measures, these being: online and offline measures. Online measures often use a think-aloud protocol, while offline measures commonly use retrospective questionnaires. Online measures are thought to assess metacognition during focal tasks, whilst offline measures assess domain-general metacognition (Ohtani and Hisasaka, 2018). Metacognitive Awareness Inventory is an example of such an offline measure. The former seems more accurate than the latter; however, the former, as online measures, can disrupt optimal task performance by eliciting reactivity in individuals by influencing strategy selection processes and exerts significantly higher mental effort than offline measures (Garner and Alexander, 1989; Zakay, 1996). Disruptions to performance can take place especially when the think-aloud protocol requires individuals to provide explanations about their thought processes rather than when they merely verbalize their thoughts during a task (Fox et al., 2011). For a task such as the Raven’s Progressive Matrices, it may be difficult for individuals to be asked to selectively report only their thought processes, and not for them to provide an explanation as to the steps they are taking to find the solutions. Therefore, the likelihood of disrupting their performance is high. In addition, thinking-aloud will increase the time taken to arrive at the solutions, and may alter the way individuals normally approaches the task. Thinking-aloud procedure has been found to interfere with performance on spatial tasks (Fox et al., 2011). Raven’s Progressive Matrices can be considered a spatial task.

Given that even a simple request for participants to provide self-confidence ratings in the accuracy of their own answers, which is considered one metacognition measure, could influence Raven’s Progressive Matrices performance (Double and Birney, 2017), the choice of measure is important. Therefore, the less impact the metacognition measure has on the task performance, the better it would be.

Setting the current study within the context of the 3-level metacognitive system described earlier (Narens et al., 1996), individuals would therefore engage in problem-solving (Object-level, L_0_). They would also utilize various strategies to enable them to problem-solve (Mid Meta-level, L_1_). Their awareness of the strategies they have used would be considered the highest level of cognition within this system (Highest Meta-level, L_2_). In the current study, a new scale will be constructed and it is best seen as operating at the highest Meta-Level L_2_. It specifically measures individuals’ awareness of their own meta-reasoning processes. To our knowledge, there are no existing scales that have attempted to measure this L_2_ Meta-Level cognition. Hence, this is a unique contribution of this research to this area of study.

Regarding individual differences in metacognitive awareness: poor performers in tasks tend to overestimate their own performance relative to good performers—known as the Dunning-Kruger effect; indeed, evidence suggests that poor performers have poorer insight, or are less aware of their own thought processes, than good performers (McIntosh et al., 2019). Moreover, people who perform poorly during analytical tasks appear less aware of their tendency to rely on intuition rather than analytical judgement, whilst those who perform well during analytical tasks show an increased awareness of their reasoning strengths and weaknesses, thus facilitating metacognitive monitoring (Pennycook et al., 2017). However, the mechanism explaining the link between insight and inaccurate self-estimation is not yet clear (Fleming and Lau, 2014). Related to self-assessment of performance and own confidence are other related concepts such as metacognition sensitivity, bias, and efficiency (Fleming and Lau, 2014). In terms of self-assessment, the current study will only examine the general idea of self-rated confidence in the accuracy of their own performance expressed as over-/under-confidence. Given the importance of the accuracy of self-report, the response bias of participants cannot be underestimated (Fastame and Penna, 2012). Therefore, this study will also examine social desirability.

Age differences have been reported in relation to Raven’s Progressive Matrices performance, in both sectional and longitudinal studies. Older adults often exhibit poorer performance on reasoning tasks than younger adults (Yuan et al., 2018). Education level is another demographic factor that has been found to relate to performance in reasoning tasks, including Raven’s Progressive Matrices (Pearman, 2020). Therefore, both age and education will be included in this study.

This study has two aims: (1) to develop a novel questionnaire to assess the awareness of individuals’ own meta-reasoning when completing the Raven’s Standard Progressive Matrices (SPM); (2) to investigate the relationship between meta-reasoning and intelligence using the newly developed Meta-Cognitive Awareness Scale-Domain-Specific, SPM standardized scores and variables such as Age, Education Level, Over-/Under-confidence and Social Desirability.

It was hypothesized that Meta-Cognitive Awareness Scale score would be positively correlated with standardized SPM scores. It was also hypothesized that Metacognitive Awareness, Age, Education level, and Over-/Under-Confidence would predict SPM scores. As such, the relationship between individual differences in factors such as metacognitive awareness, and ability as exhibited in their SPM performance could be explored.

## Materials and Methods

### Participants

There were 100 participants, however, two participants were excluded due to the loss of most of their data. The remaining 98 participants consisted of 68 females and 30 males. Overall mean age was 33.1 (*SD* = 16.5); age range: 18–79 years. Most participants (51%) had completed their high school education, and 59.32% of participants were students. Only 36.7% of participants claimed to have had previous experience with Raven’s Progressive Matrices or other similar tasks.

### Procedure

This study was approved by the Federation University Human Research Ethics Committee. All participants provided written informed consent and administered the 60-item SPM in paper-and-pen format using standardized instructions with no time limit. They then completed the Meta-Cognitive Awareness Scale – Domain Specific (MCAS-DS) and accompanying items exploring participants’ experiences immediately after they had completed the SPM to help them to more easily recall their experience and strategy and thus minimize recall failure. The Marlowe-Crown Social Desirability Scale was then administered. Some basic demographic information, including their highest education level were also obtained. For a smaller group of participants, they were also administered the Metacognitive Awareness Inventory (MAI) and Rotter’s Locus of Control scale. Unfortunately, there was a technical error during the data-collection process whereby only a small subgroup of participants (*n* = 16, 6 males and 10 females) were administered the additional two scales.

### Raven’s Standard Progressive Matrices (SPM)

All 60 items of the SPM (Raven et al., 2000) were used. Each SPM item was presented as a puzzle in a matrix format, from which a piece had been removed. Either six or eight possible solution pieces, from which only one piece correctly completed the matrix, were offered. The SPM has good psychometric properties with test-retest reliability coefficients ranging from 0.83 to 0.93 and construct validity coefficients ranging from 0.81 to 0.94 (Raven et al., 2000). Only correct items were tallied, and the scores transformed into standardized scores.

### Meta-Cognitive Awareness Scale – Domain Specific (MCAS-DS): Scale Development

Given the aim of this study is to examine meta-cognitive awareness when performing SPM, and no such domain-specific offline questionnaire exists, a scale which focuses on Raven’s Progressive Matrices was constructed. Although the structure of other metacognitive questionnaires such as the Metacognitive Awareness Inventory (MAI) were used as an initial reference, the phrasing of the items were distinctively different to those on other questionnaires such that this current scale refers specifically to Raven’s Progressive Matrices, and uses words like puzzles and matrix to focus attention on the task at hand, and not on other generic tasks. From the initial larger pool of items, 40 items that the researchers deemed to be relevant and clearly covering the conceptual scope of metacognitive awareness were selected for further refinement. Half of these were positively and negatively worded items. Items included: ‘I do not slow down when I encounter important information about the puzzles’, ‘I know how to modify a strategy if it is not helping me to solve a puzzle’, and ‘I find myself pausing regularly to check my comprehension of the information presented in a puzzle’. All items were presented on a 5-point Likert scale ranging from Strongly Disagree to Strongly Agree, unlike the 100-mm bipolar scale used in the MAI. All the items that were negatively worded were reverse-scored such that the higher the total score, the better the meta-cognitive awareness of the individual.

Discriminatory Index - Extreme Group method as described by McIntire and Miller (2000) was adapted for use here to ensure that each item can discriminate between those who have high overall scale scores and those who have low overall scale scores. The highest 25% scorers and lowest 25% total MCAS-DS scorers’ item scores were first identified. For each of the 40 items, the difference between the proportion of individuals in each group who scored 4 (Agree) and 5 (Strongly Agree) were calculated. Items that differentiated high total scorers from low total scorers (D = 0.30) were retained, while items that did not adequately differentiate high scorers from low scorers were excluded. By doing so, the scale was reduced to 18 items. These items were therefore most effective in discriminating the highest scorers from the lowest scorers. The scale was further refined and examined using Exploratory Factor Analysis (EFA), the results of which will be reported in the Results section. A 15-item scale was finally derived and utilized for subsequent explorations.

In addition to the Likert scale items, eight short additional questions that explored participants’ performance and experience of the Raven’s Standard Progressive Matrices (SPM) were constructed. These included questions such as whether participants had previous experience with the SPM, whether they think they can improve their own performance, and their level of confidence regarding their responses. This last item was measured using a 7-point Likert scale. One of the questions also required participants to endorse as many of the listed strategies they used to solve the SPM. These strategies were derived from descriptions of the SPM from the test manual and literature (Prabhakaran et al., 1997). They include, ‘Looking for how 3 characteristics such as 3 geometric shapes or 3 line textures are distributed through a row’ and ‘Verbally repeat some characteristics of a figure to help solve the puzzles’. Also included on the list of strategies was one improbable strategy (‘Associating even numbers to shapes and then perform calculations’). This was included as a mean to verify whether participants would endorse it either due to a tendency to acquiesce, or perhaps exhibit social desirability by endorsing as many strategies as possible.

### Marlowe-Crown Social Desirability Scale

To control for response bias, social desirability was measured via a short 13-item True-False version of the Marlowe-Crown Social Desirability Scale (Reynolds, 1982). Example item reads: ‘I sometimes feel resentful when I don’t get my way.’ This scale displays acceptable internal reliability of 0.76, and compares well to the standard version (Reynolds, 1982). Test-retest reliability was 0.74 and the scale demonstrated good concurrent validity of 0.93 with the standard form (Reynolds, 1982; Zook and Sipps, 1985). High score suggests a tendency to appear more socially desirable and thus more likely to complete items in order to avoid disapproval of others.

### Metacognitive Awareness Inventory

The Metacognitive Awareness Inventory (Schraw and Dennison, 1994, [MAI]) was utilized for a sub-group of 16 participants to examine its relationship with the current domain-specific metacognitive awareness questionnaire. It is postulated that the 2 scales would be different as they measure metacognitive processes at different levels, and that MAI assesses metacognitive awareness associated with learning, whilst the MCAS-DS assesses metacognitive awareness specifically related to Raven’s Progressive Matrices. MAI contained 52 items using a 100-mm bipolar scale. However, as it is not central to the current study, the response format of a dichotomous scale was used instead.

### Locus of Control Scale (Rotter, 1966)

Locus of Control Scale consists of 29 forced-choice items which included 6 filler items. This was administered to only 16 participants due to a technical error made during data collection process. It measures generalized belief in internal-external control of events in life. Higher scores are indictive of the tendency of the individual believing that life events are contingent on external factors such as luck and environment.

### Education Level

Education level was measured by asking participants to indicate their highest educational qualification, ranging from 1 (primary school), through to postgraduate qualifications (5).

## Results

### Meta-cognitive Awareness Scale – Domain Specific (MCAS-DS) Scale Development

Exploratory Factor Analysis was undertaken to examine the initial 18-item scale which was derived after the Discriminatory Index method, as described earlier, was applied. Following Field’s (2013) analysis procedure, the Kaiser-Meyer-Olkin (KMO) statistics was examined together with the diagonal element of the anti-image correlation matrix. Two items (22 and 1) with values below 0.50 in the latter were removed as recommended. The final KMO value (0.61) is mediocre (Kaiser, 1974). Bartlett’s Test of Sphericity reached statistical significance indicating that chosen analysis method would be suitable. Initial analysis with Maximum Likelihood extraction showed seven factors with eigenvalues above 1, and they each explained a range of the observed variance, decreasing from 15.54% for the first factor down to 6.48% for the seventh factor. The scree plot did not show an obvious break which would have enabled a clear decision of the number of factors to be retained.

When the analysis was conducted again with the 16-item scale, 6 factors were extracted, cumulatively explaining 62.83% of variance. Given that the factors were intercorrelated, even if only weakly, Direct Oblimin rotation method was used. The Pattern Matrix showed that there was one factor with only 2 items, one of which also loaded onto another factor. Therefore, the lone item (18) was removed. This resulted in a 5-factor solution using Kaiser’s criterion, and improved the interpretability of the Pattern Matrix. This 5-factor solution accounted for 40.91% of the variance after rotation. The Chi-square goodness-of-fit index, showed this to be a good fit [χ^2^ (40) = 25.88, *p* = 0.96] although it is acknowledged that this goodness-of-fit index is sensitive to sample size (Anderson and Gerbing, 1984). Therefore, the final scale consisted of fifteen items and five factors were retained. Table 1 shows the factor loadings of the final 15-item scale after rotation.

**TABLE 1:** Rotated Factor Loadings for the MCAS-DS (*N* = 98).

		Rotated factor loadings				
	Item	Awareness of engagement in self-monitoring	Awareness of own ability	Awareness of response speed/time	Awareness of alternative solutions	Awareness of requisite problem-solving resources
21	I find myself pausing regularly to check my comprehension of the information presented in a puzzle.	**1.028**	0.071	–0.147	0.115	0.023
6	I do not slow down when I encounter important information about the puzzles.	**0.442**	–0.052	0.305	–0.081	0.003
28	I know how to modify a strategy if it is not helping me to solve a puzzle.	0.081	**0.944**	0.108	–0.074	–0.144
11	I can identify the most important information about the puzzles.	–0.076	**0.392**	0.071	–0.030	0.076
26	I use my intellectual strengths to compensate for my weaknesses.	0.034	**0.384**	–0.076	0.089	0.146
9	I do not often check my understanding of significant information about a matrix.	–0.028	0.180	**0.687**	0.035	–0.058
4	When I am unsure about the correct answer to solve a matrix I rarely hesitate.	0.002	–0.180	**0.524**	0.048	0.118
20	I do not stop and review a puzzle when I get confused.	0.056	0.151	**0.466**	0.050	0.102
33	I find myself evaluating how many possible answers I have narrowed down to determine my progress in solving a puzzle.	–0.110	0.060	–0.057	**0.647**	0.017
35	I do not ponder for a long time over two answers that could possibly solve the puzzle.	0.174	–0.228	0.114	**0.562**	0.034
16	I think of several ways to solve a puzzle and choose the best one.	0.111	0.141	0.163	**0.314**	–0.103
34	Information about a puzzle that is simple will be easier to comprehend than information that is complicated.	–0.088	0.115	–0.141	0.102	**0.586**
24	Information about a puzzle that is straightforward will be more time consuming than information that is complicated.	0.071	–0.080	0.246	–0.247	**0.575**
39	I am unaware of the strategies I use when solving puzzles on the Raven’s Progressive Matrices	–0.151	0.078	0.131	0.117	**0.432**
29	I do not think that some people will have more intellectual weaknesses than others in solving the Raven’s Progressive Matrices	0.094	–0.009	0.031	–0.049	**0.322**
	Initial Eigenvalues	2.593	1.972	1.699	1.318	1.109
	% of variance after extraction	9.803	10.192	10.129	5.969	4.820

*Items loading strongest to each factor appear in bold.*

The first factor of this 15-item scale explains 9.80% of the variance, and encompasses two items. It consists of items describing Awareness of Engagement in Self-monitoring (e.g., ‘I find myself pausing regularly to check my comprehension of the information presented in a puzzle’. The second factor of three items, explains 10.19% of variance, and it relates to Awareness of Own Ability (e.g., ‘I know how to modify a strategy if it is not helping me to solve a puzzle’). The third factor, Awareness of Response Speed, explains 10.13% of variance and has items including ‘When I am unsure about the correct answer to solve a matrix, I rarely hesitate.’ The fourth factor explains 5.97% of the variance, whilst the fifth factor explains 4.82% of the variance. The fourth factor, Awareness of Alternative Solutions, contain items such as ‘I find myself evaluating how many possible answers I have narrowed down to determine my progress in solving a puzzle’. Whilst the final factor, Awareness of Requisite Problem-Solving Resources, contains items such as ‘Information about a puzzle that is straightforward will be more time consuming than information that is complicated’. In terms of the scale reliability, Cronbach’s alpha coefficient for the full 15-item scale was 0.63.

To examine the MCAS-DS properties, correlation analysis was also conducted for a subgroup of 16 participants. The subgroup analysis using a very small sample here is not ideal and stemmed from a technical error at the point of data collection. No significant relationship was found between MCAS-DS and the MAI (*r* = 0.194, *n* = 16, *p* > 0.05; Percentile Bootstrap 95% CI [−0.361, 0.689]). Confidence interval was calculated using bootstrapping technique. Similarly, no significant correlation was found between MCAS-DS and Locus of Control (*r* = −0.295, *n* = 16, *p* > 0.05; Percentile Bootstrap 95% CI [−0.714, 0.172]). It is plausible that the lack of significant correlations here are due to the small sample size, thus restricting the data range which is problematic when conducting correlational analysis (Bland and Altman, 2011). As previously postulated, there is, however, no expectation that there should be significant relationship between scales that measures meta-cognitive processes operating at different levels, nor with unrelated scales such as Locus of Control. Together, despite the sample size limitation, it may indicate the discriminant validity of the MCAS-DS. However, this will need further exploration in future studies.

### Descriptive Statistics for Variables

Means, minimum and maximum scores, and standard deviations of other variables in the study are shown in Table 2.

**TABLE 2:** Means, minimum and maximum scores, and standard deviation (SD) of the variables.

	Mean	*SD*	Minimum	Maximum	n
Standardized SPM	106.40	14.32	66	137	97
Metacognitive Awareness (MCAS-DS)	52.69	5.51	38	67	98
Age	33.09	16.51	18	79	97
Education Level	2.68	0.94	1	5	97
Social Desirability	6.20	3.03	0	13	93
Number of strategies reported	6.62	1.96	1	11	98
Level of confidence in accuracy of own answers	4.69	1.33	1	7	97
Over-/Under-Confidence	0.00	1.08	−2.98	1.99	98
Metacognition Awareness Inventory (MAI)	33.63	5.24	27	42	16
Locus of Control	12.31	2.73	9	17	16

As part of the general exploration of participants’ experience relating to SPM, participants were presented with a few other SPM test-taking behavior items and their responses are summarized in Table 3. The majority of participants (63.3%) have not had experience with SPM and thought they could improve their own performance. In terms of strategies, the majority of participants (66.3%) said they have used different strategies to solve different SPM items instead of using the same one. Lastly, the researchers constructed an improbable strategy and embedded it amongst other strategies that could be used to solve SPM items, to which, only a minority (30.6%) endorsed it.

**TABLE 3:** Frequencies of responses for items relating to SPM experience.

	Yes (%)	No (%)	n
Had previous experience with SPM or SPM-like task	36 (36.7)	62 (63.3)	98
Whether or not own SPM performance could have been improved	65 (66.3)	33 (33.7)	98
Used the same strategy for all SPM items	25 (25.5)	72 (73.5)	97
Used an “improbable” strategy to solve SPM	30 (30.6)	68 (69.4)	98

### Confidence and Over-/Under-Confidence

Participants’ confidence in the accuracy of their own SPM answers was significantly correlated with their SPM performance (*r* = 0.420, *n* = 96, *p* < 0.01). Over-/Under-Confidence was derived by subtracting the SPM z-score from the level of confidence z-score. Therefore, the closer the Over-/Under-Confidence score is to zero, the more accurate the individual is in rating their own level of confidence that is commensurate with their performance level. Positive score indicates Over-confidence and negative score indicates Under-confidence.

### Metacognitive Awareness and SPM

A significant positive correlation was found between Metacognitive Awareness and SPM standardized scores, *r* = 0.295, *n* = 97, *p* = 0.003, such that the higher the MCAS-DS scores, the higher the SPM standardized scores.

### Metacognitive Awareness, Age, Education Level, Over-/Under-confidence, Social Desirability and SPM

A standard Enter method multiple regression was conducted to examine the relationship between Metacognitive Awareness, Age, Education level, Over-/Under-confidence, Social Desirability and standardized Raven’s SPM. The correlations between the variables are presented in Table 4.

**TABLE 4:** Intercorrelations of SPM, Metacognitive Awareness, Age, Education Level, Over-/Under-confidence, and Social Desirability.

	1	2	3	4	5
1. SPM scores	-				
2. Metacognitive Awareness (MCAS-DS)	0.295**	–			
3. Age	0.016*ns*	−0.102 ns	–		
4. Education Level	0.173*	−0.108 ns	0.038 ns	–	
5. Over-/Under-confidence	−0.538**	−0.023 ns	−0.152 ns	−0.154 ns	–
6. Social Desirability	−0.082*ns*	−0.109 ns	0.247**	−0.113 ns	−0.039 ns

ns = not significant (p > 0.05), *p < 0.05, **p < 0.01 (2-tailed).

**FIGURE 1:**
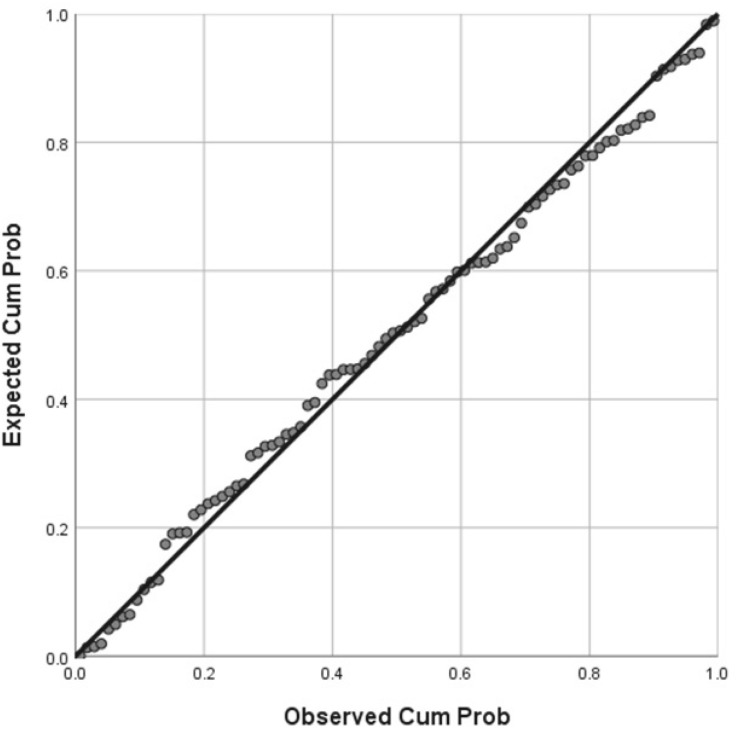
Normal P-P Plot of Regression Standardized Residual where the Raven’s Progressive Matrices standardized scores was the dependent variable.

**FIGURE 2:**
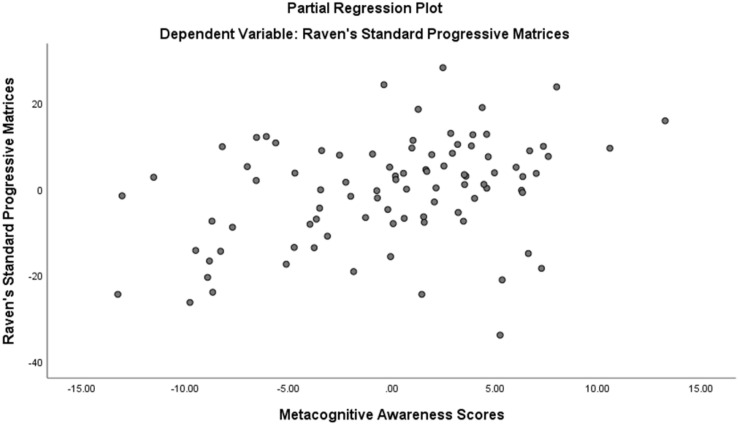
Partial Plots of Raven’s Progressive Matrices against Metacognitive Awareness.

**FIGURE 3:**
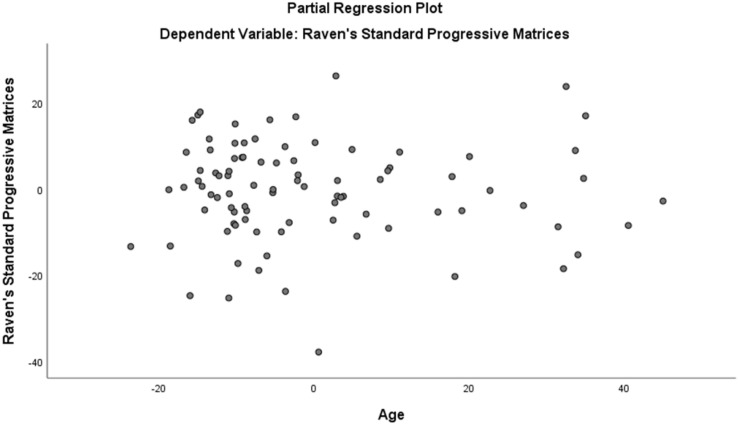
Partial Plots of Raven’s Progressive Matrices against Age.

**FIGURE 4:**
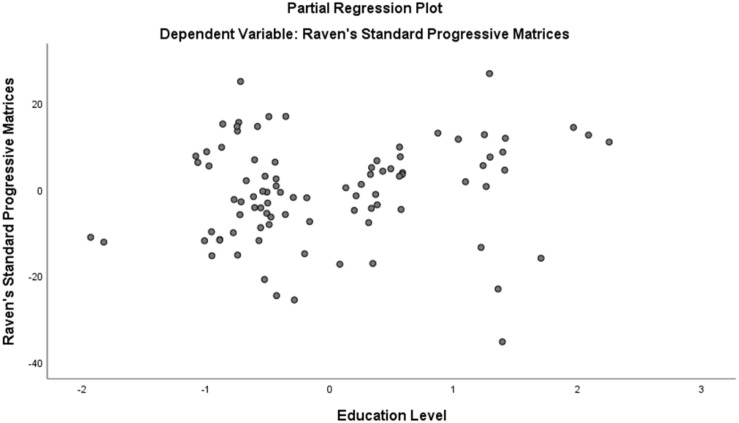
Partial Plots of Raven’s Progressive Matrices against Education.

**FIGURE 5:**
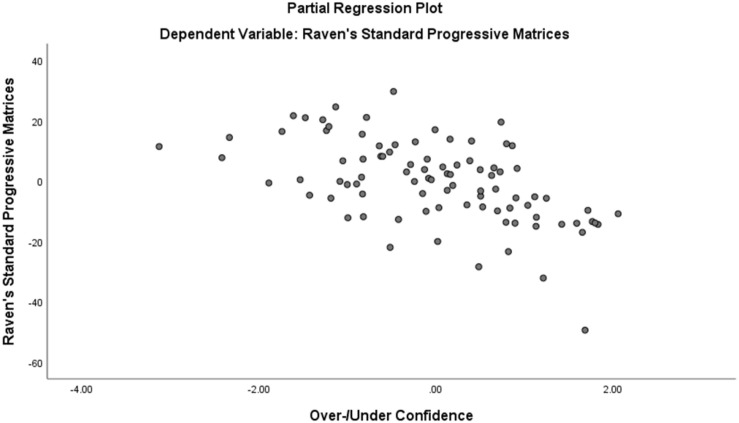
Partial Plots of Raven’s Progressive Matrices against Over-/Under Confidence.

**FIGURE 6:**
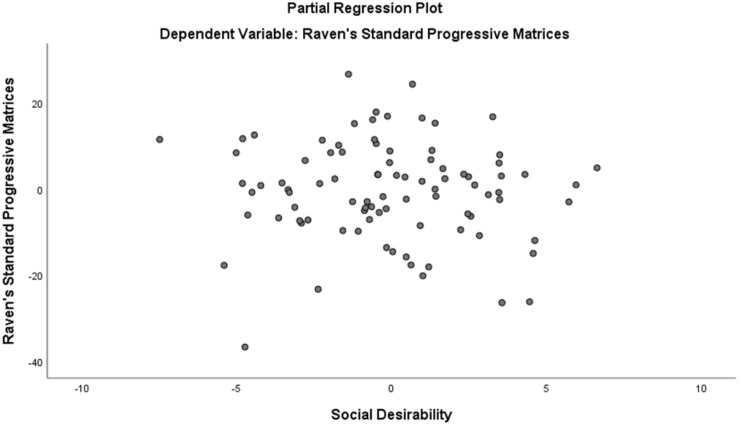
Partial Plots of Raven’s Progressive Matrices against Social Desirability.

Assumption of multicollinearity was not violated as indicated by variance inflation factor analyses (VIF) indices being less than 2 for all variables (VIF ≥ 1). No violation of the normality, linearity and homoscedasticity were found. These were checked by inspecting the Normal Probability Plot of the Regression Standardized Residual which showed that the points lie in a relatively straight diagonal line with no major deviations from normality (See Figure 1). The scatterplot of the standardized residuals did not show any clear pattern in their distribution (See Figures 2–6). There were no outliers detected from the scatterplot nor from examining the Mahalanobis distances values where none of the cases exceeds the critical value of 20.52. Durbin-Watson statistics (2.121) showed that adjacent residuals were uncorrelated, hence independence of errors can be assumed.

**FIGURE 7:**
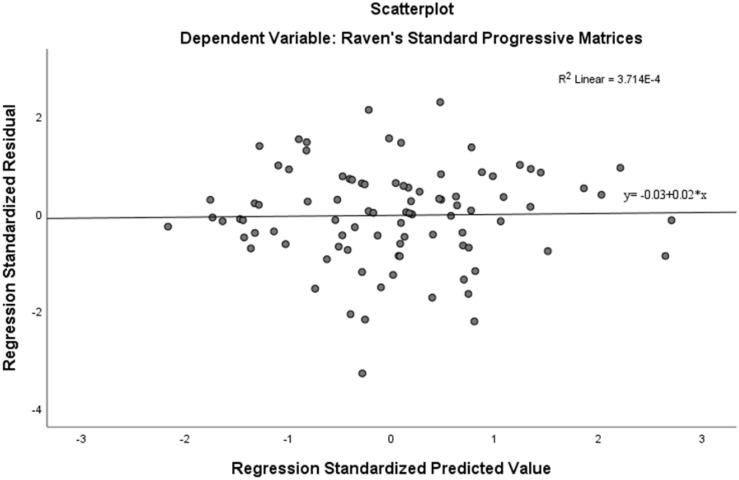
Scatterplot of standardized predicted values against standardized residuals.

Together, Metacognitive Awareness, Age, Education Level, Over-/Under-confidence and Social Desirability as a model, significantly predicted the standardized SPM scores *F*(5, 85) = 10.831, *p* < 0.001; and explained 38.9% of the variance of standardized SPM. *R*^2^ = 0.389 (Adjusted *R*^2^ = 0.353). Cohen’s *f*^2^ = 0.637 indicated that this is a large effect size. See the plot of standardized predicted values against standardized residuals (Figure 7).

Upon closer examination of the individual predictors (Table 5), it was found that two variables were significantly and uniquely making contribution. They were the Over-/Under-confidence measure, and the metacognitive awareness measure. For the Over-/Under-confidence measure (*β* = −0.519, *t*(85) = −5.977, *p* < 0.001), it was negatively associated with SPM scores. Specifically, the more under-confident the individuals, the better they performed on SPM. Metacognitive Awareness measure scores [*β* = 0.288, *t*(85) = 3.340, *p* = 0.001] were positively associated with SPM scores such that individuals with higher meta-cognitive awareness tended to also score higher on SPM.

**TABLE 5:** Linear model of predictors of standardized Raven’s SPM, with 95% confidence intervals in parentheses.

Model		*b*	*SE B*	*β*	*p*
**1**	**(Constant)**	64.273 (37.086, 91.460)	13.674		*p* < 0.001
	**Meta-cognitive Awareness**	0.748 (0.303, 1.194)	0.224	0.288	*p* = 0.001
	**Age in years**	−0.022 (−0.175, 0.132)	0.077	−0.025	*p* = 0.780
	**Education Level**	1.817 (−0.820, 4.453)	1.326	0.119	*p* = 0.174
	**Over-/Under-confidence**	−6.871 (−9.157, −4.585)	1.150	−0.519	*p* < 0.001
	**Social Desirability**	−0.243 (−1.076, 0.590)	0.419	−0.051	*P* = 0.564

*R^2^ = 0.389 (p < 0.001).*

## Discussion

This study investigated the relationship between metacognitive awareness and standardized SPM scores that are most often linked to problem-solving and intelligence. To enable the measurement of metacognitive awareness, a domain-specific, Raven’s SPM-focussed questionnaire was constructed, and then administered immediately following the completion of the focal task. As an offline measure, the construction of the current scale addresses the often-cited problems with online metacognition measurements which are, the disruption of optimal task performance, and the reactivity exhibited when eliciting metacognitive information (Double and Birney, 2019). In addition, there is the issue of metacognitive questionnaires being too generic and not reflecting the cognition involved in the specific task (Allon et al., 1994). This approach of constructing a measure based on the task used (e.g., SPM) has not yet been adopted in this field of research and therefore this study presents for the first time, a novel approach to the investigation of meta-cognitive processes.

The first aim of this study was to develop a domain-specific meta-cognitive awareness questionnaire. A 15-item scale was developed and analyzed using Exploratory Factor Analysis. A five-factor solution emerged; these components are named as ‘Awareness of Engagement in Self-Monitoring,’ ‘Awareness of Own Ability,’ ‘Awareness of Response Speed/Time,’ ‘Awareness of Alternative Solutions,’ and ‘Awareness of Requisite Problem-solving Resources.’

The first factor captured participants’ awareness that they are engaging in the monitoring their own processing of the test items, such that they know they are pausing to check their own comprehension. This may be related to the top level cognition in the proposed 3-level metacognitive system (Narens et al., 1996), where participants are not only aware but monitor the strategies they are using, in addition to utilizing strategies and engaging in problem-solving.

The second factor, Awareness of Own Ability, reflects participants’ knowledge and perhaps confidence that they know how to solve the items by modifying their initial strategy to replace an unsuccessful one. The emergence of the third factor relating to response speed or time is unexpected because the SPM was administered without any time-limit. There may be different explanations for this. Processing speed has been often cited as an important component of intelligence (Schubert and Frischkorn, 2020), and perhaps reflects the implicit theories laypersons have of intelligence (Langfeldt and Imhof, 2001). Therefore, it may also reflect participants’ implicit theory that the speed of responding may be important, despite no time-limit being imposed on them. After all, many tests require test-takers to complete as many items as possible under a set time limit. It is also possible that participants are aware that there are a series of items that required their attention, hence they self-imposed a limit on the time they spend on solving each item in order to complete all the items.

The fourth factor, Awareness of Alternative Solutions, relates to participants showing their awareness that there may be other seeming possible solutions to the puzzles and therefore monitoring these will enable them to arrive at the best solution. As for the final factor, Awareness of Requisite Problem-Solving Resources, it consists of participants being aware of their possession of requisite knowledge when solving problems. For example, a longer time may need to be allocated to solving puzzles with complex information. It is perhaps akin to how learners allocate their effort during learning which is part of metacognitive monitoring judgements (Baars et al., 2020).

Relating this to the 3-level metacognitive system (Narens et al., 1996) the participants therefore engage in problem-solving (Object-level, L_0_) of the SPM, utilizing various strategies to enable them to problem-solve (Mid Meta-level, L_1_), such as changing approach to problem-solve, but they are also aware of their own engagement in self-monitoring, their own ability, the response speed or time they utilize, that there may be alternative solutions, and the necessary ingredients for them to solve the puzzles (Highest Meta-level, L_2_). This 3-level metacognitive system seemed to provide the best theoretical, *a priori* fit to the results here. However, it is recognized that this is an open empirical question as there are other models which describes the different relationships between the information used for problem-solving and those used for metacognitive self-evaluation (e.g., Maniscalco and Lau, 2016; Fleming and Daw, 2017).

In terms of the psychometric properties of the 15-item Meta-Cognitive Awareness Scale – Domain Specific (MCAS-DS), Cronbach’s alpha reliability was relatively low (*α* = 0.63 for the full 15-item scale). Ideally, this should be higher and therefore, further refinement of the scale would be necessary. The correlation between MCAS-DS and the Metacognitive Awareness Inventory (MAI) is not statistically significant. There may be a few reasons here – firstly, this may not be surprising given that the former has been constructed to specifically focus on one task, the SPM, whilst the MAI is typically used in a generic learning-related context. Secondly, although bootstrapping of the correlational analysis was undertaken, the restriction in terms of available MAI data remains a limitation for the analysis, and should be addressed in future studies. Conceptually, the MCAS-DS is deemed to measure the awareness of strategies participants used, thus operating at the Meta-Level L_2_ as described by Nelson (1996) as opposed to MAI that did not specify the level it measured. No significant relationship was found between MCAS-DS and Locus of Control, and this is an additional indication of the discriminant validity of the MCAS-DS.

Given that this study may be the first and the only exploration of a domain-specific questionnaire linked closely to the focal task to date, it warrants further examination and development to improve its psychometric properties. This approach to the construction of a domain or task-specific task, however, is unique. It also fits well with literature that posits the importance of task-specific measures (Thompson, 2000).

Although some might question the usefulness of such a specific metacognitive awareness questionnaire, it is worth noting that Raven’s Progressive Matrices, as a family of ability tests, are closely aligned to fluid intelligence and reasoning; hence, further examination may yield new information about fluid intelligence and reasoning. In addition, the matrices have properties that could lend themselves to segmentation of cognitive processes—a strength already recognized in the field of cognitive and neuroimaging studies (Carpenter et al., 1990; Prabhakaran et al., 1997; Christoff et al., 2001; Silberstein et al., 2004; Song, 2005). Chuderski et al. (2020) summarizes the types of methods used to examine the cognitive processes involved in solving the matrices. They include identification of strategies using eye-tracking technique, and examining elementary cognitive variables such as working memory capacity. These demonstrate the utility and level of interest researchers have in understanding the matrices. This therefore merits the pursuit of dedicated, domain-specific meta-cognitive scales to help segment the respective cognitive process that underpin higher-order thinking, and so advance understandings of fluid intelligence.

Metacognitive awareness was hypothesized to correlate positively with SPM standardized scores, and indeed a significant positive correlation was observed. This agrees with previous research that showed a positive correlational relationship between metacognition and intelligence (Veenman and Beishuizen, 2004). The strength of the current metacognitive awareness-intelligence relationship is comparable to that reported in a recent meta-analytic study (Ohtani and Hisasaka, 2018) even though the current study utilized a domain-specific metacognitive awareness measure. This should not diminish the importance of the current measure given that it addresses important theoretical and measurement issues that will be discussed further later.

Previous research showed that higher metacognitive knowledge was displayed more in gifted children in comparison with children of high-average and low-average intelligence (Swanson, 1992). Campione and Brown (1978) even attributed differences in intelligence level to the variations in the efficiency of executive or metacognitive processes. The current finding also supports the Triarchic Theory of Intelligence (Sternberg, 1988), which postulates a close relationship between metacognition and intelligence, even if the exact metacognition-intelligence relationship and direction of causality is currently unclear.

Contrary to current findings, Allon et al. (1994) found a non-significant relationship between metacognition and intelligence. Allon et al. speculated that if the problem-solving task that they employed in their study of metacognition has been more closely related to an intelligence test, then, perhaps a metacognition-intelligence relationship could have been found. This study did precisely address Allon et al.’s (1994) point by using the SPM as a measure of intelligence and as the focal task upon which participants’ metacognitive awareness was measured. Hence, this novel method may have contributed to the significant relationship found between metacognitive awareness and intelligence here. It would appear that similar to others’ finding that reasoning processes are task- and content-specific, rather than domain-general (Thompson, 2000), a task-specific scale such as the current MCAS-DS is necessary for the study of metacognitive awareness during SPM.

To achieve the second aim of this study to examine the relationship between metacognitive awareness and other variables with SPM, a multiple regression was conducted. Specifically, the hypothesis was that SPM scores would be predicted by Metacognitive Awareness, Age, Education Level, Over-/Under-confidence and Social Desirability. The model containing the chosen variables predicted SPM and explained 38.9% of the SPM variance. Of the variables examined, only Over-/Under-confidence and Metacognitive Awareness significantly predicted SPM independently.

Higher Metacognitive Awareness scores were associated with higher SPM scores. This seemed to indicate that factors such as being aware of one’s own engagement in self-monitoring, own ability and so on, are related to SPM performance. The more aware the individuals are of these, the better their performance. Over-/Under-confidence also significantly predicted SPM such that the more over-confident (positive value) the participant, the lower their SPM performance.

The ability to evaluate one’s own performance has previously been studied using an online metacognition measure of self-rated confidence administered after every item of SPM. Those who displayed higher meta-cognitive awareness were found to perform better than those who did not (Double and Birney, 2017). In this study, rating of self-confidence was elicited offline following the completion of the whole SPM task. It also showed a similar performance outcome as seen in other studies wherein self-confidence is positively correlated with SPM scores. This self-confidence was then used to calculate the Over-/Under-confidence score.

This Over-/Under-confidence score provided an additional metacognitive measure at the mid meta-level (L_1_) of the 3-level metacognitive system. This measure incorporated the subjective self-evaluation with the more objective actual performance. It indexes a person’s self-rated confidence that is linked to their actual performance. Therefore, it is not just about a person’s self-confidence but whether that confidence level is commensurate with their performance level. According to the results, being able to have the level of confidence that is commensurate with their performance appeared to be a factor that is linked to better performance. This is akin to measuring the “accuracy” of their confidence level instead of having misplaced confidence.

In this study, individuals who are over-confident with their performance and whose confidence are not justified by their actual performance showed lower SPM scores. This fits well with the Dunning-Kruger effect that poor performers seemed to have less insight, or in this case, less “accuracy” in their assessment of their own thought processes. Considering both Over-/Under-confidence measure and self-confidence measure, it appeared that having self-confidence may be good but only if it is commensurate with their level of task performance.

At the higher meta-level, L_2_ where the current MCAS-DS operates, a positive relationship between metacognitive awareness and SPM was found. This is another indication that the more aware individuals were of their meta-reasoning processes, the better their performance on reasoning tasks such as SPM. This agrees with previous findings of Dunning-Kruger effect in high level reasoning whereby analytic thinkers, rather than intuitive thinkers, were found to be more aware of their reasoning strengths and weaknesses (Pennycook et al., 2017). Further research is necessary to explain how participants’ insight relates to their overestimation (Dunning-Kruger effect) or under-estimation (intellectual humility) of their own performance (Krumrei-Mancuso et al., 2019). The relationship between self-confidence and performance also warrants further investigation much like other studies conducted in the field of perceptual confidence and metacognitive awareness on visual working memory (Samaha et al., 2016). In addition, there are findings that suggest that there may be sex difference in confidence and metacognitive monitoring accuracy for ability measures, especially in the spatial abilities domain. Although females displayed lower confidence in their monitoring and assessment of their overall or global performance compared to males, their actual performance on the task did not differ from those of their male counterparts. What is interesting is that females, in their trial-by-trial performance monitoring, as opposed to their global performance monitoring, showed that their own performance monitoring was accurate. It may be that females utilize strategies that are different from those used by males when solving spatial problems. They may be also utilizing cues differently (Ariel et al., 2018). Further scrutiny of sex difference in confidence ratings in future studies using a spatial task such as Raven’s Progressive Matrices may be helpful.

In this study, although education level was correlated with Raven’s Progressive Matrices performance, it did not significantly predict the performance. Age did not correlate nor predict Raven’s Progressive Matrices performance. These do not agree with previous findings. The reason for this may be partly due to the relatively smaller sample size of the current study, and the different age groups examined in this study and previous studies (Pearman, 2020). Mean age of participants in previous studies was higher than the mean age of participants in this study (Tucker-Drob et al., 2014; Yuan et al., 2018). These could be further examined with a sample that has better distribution of age, and perhaps education level too, given that over half of the participants in the current study completed high school only. It is noted that approximately 37% of the participants have experience with Raven’s Progressive Matrices or similar items and it is possible that this might have an influence on their performance and their awareness of their own Raven’s problem-solving strategy.

The strength in this study stems from the approach taken to the study of metacognitive awareness and embedding it within the 3-level metacognitive system. The construction of the MCAS-DS, a domain-specific meta-cognitive awareness measure, that is directly linked to the focal task and yet minimizes disruption to task performance by it being an offline measure, is unique. It addressed issues raised in previous research, including that reasoning tasks are governed by their own unique parameters (Allon et al., 1994; Thompson, 2000), and therefore require exploration using their own parameters. SPM, as an often-used reasoning task which is also used for the study of intelligence, has therefore its own unique parameters that are best understood using a task-specific scale. This MCAS-DS minimizes disruption to, and impact of, metacognition measure on task performance, and also minimizes reactivity in participants in its design by being an offline measure (Garner and Alexander, 1989; Zakay, 1996; Double and Birney, 2019).

There are a number of ways in which this study could be improved. The scale’s psychometric properties will need further refining. It may involve adding additional items and improvement in the wording of the items to improve its reliability and construct validity. The latter will ensure that all aspects relating to the awareness of their SPM-taking behaviors is captured as comprehensively as possible. Further validation of the scale using a larger sample, and using it with other meta-cognitive scales is recommended. A comparison between how well this scale performs against other scales will be helpful even though the current scale already addresses important theoretical (Thompson, 2000) and measurements issues (Garner and Alexander, 1989; Zakay, 1996; Double and Birney, 2019) not addressed by other known generic metacognitive scales. The sample utilized in the current study encompasses a broad range of individuals of different ages and background but it is less than ideal in terms of sample size. Further validation of the scale using a much larger sample size and using different sample groups would be necessary.

Whilst the current results showed that participants who performed better also tended to show higher metacognitive awareness scores, all the participants were administered the same task without controlling for task difficulty. It is possible that lower metacognitive scores may be attributed to the participants’ “struggle” with completing the Raven’s Progressive Matrices. Therefore, they were unable to complete the metacognitive scale well as a result of that, rather than due to their poorer metacognitive awareness. There may be steps that can be undertaken to overcome this. For example, it is possible to pre-test participants to determine the proportion of participants who can complete each test items successfully, and then select the items that are deemed to be of a particular difficulty level for subsequent administration to other participants. This then can be used to control for task difficulty. An example of this is found in Ackerman (2014). This can be examined in future studies.

There may be other confounding factors that may influence the results here, for example, social desirability as well as other types of response bias. In the current study, social desirability, was included in the multiple regression analysis but did not show a significant relationship to the Raven’s Progressive Matrices scores. There were also no significant relationship between social desirability and metacognitive awareness. Other types of bias, for example, extreme responding or tendency to respond using only particular section of the scale are more difficult to control. However, these response bias issues can, and should be investigated further (Kreitchmann et al., 2019).

In addition to the above suggestions of improvement and future exploration, as an extension of the current study, it may soon be possible to link the present findings to the findings of brain imaging findings so to further elucidate the relationship between brain function in metacognitive awareness and intelligence. Currently however, brain imaging research continues to present divergent results and theories for understanding the neural basis of metacognition; indeed, one study suggests that metacognitive processes are underpinned by distinctive neural substrates (Valk et al., 2016), whereas another study posits the existences of broader task-dependent, domain-specific and domain-general networks (Rouault et al., 2018); and a third study posits frontoparietal networks as always being involved in metacognition regardless task or judgment type (Vaccaro and Fleming, 2018).

## Conclusion

In conclusion, this study uniquely employed the offline recall method with a domain-specific metacognitive awareness measure to assess the relationship between Metacognitive Awareness and Intelligence, and further examined the role of Social Desirability and Over-/Under confidence during problem-solving task of Raven’s SPM. This approach to scale-construction in this field addresses often-cited concerns with online metacognition measures whilst providing opportunities to examine test-taking behaviors during Raven’s SPM. There is a need to improve the metacognitive awareness measure and to seek further opportunity to explore the different meta-level processes. The use of a larger sample size and with a different sample to ensure replicability of the findings will also be necessary. However, take together, this paper describes an example of a potentially fruitful approach to the construction of future metacognitive measures.

## Data Availability Statement

The raw data supporting the conclusions of this article will be made available by the authors, without undue reservation.

## Ethics Statement

The studies involving human participants were reviewed and approved by Human research ethics committee, Federation University. The patients/participants provided their written informed consent to participate in this study.

## Author Contributions

JS contributed to the conception, design of the study, and performed the statistical analysis. SL and BL contributed to the writing and editing of earlier drafts of the manuscript. All authors contributed to manuscript revisions, read and approved the submitted version.

## Conflict of Interest

The authors declare that the research was conducted in the absence of any commercial or financial relationships that could be construed as a potential conflict of interest.
